# Supplementary material for: Lisa: inferring transcriptional regulators through integrative modeling of public chromatin accessibility and ChIP-seq data
Source: Genome Biol. 2020 Feb 7;21:32. doi: 10.1186/s13059-020-1934-6 (PMC7007693; doi:10.1186/s13059-020-1934-6)
Supplement: Supplementary file 1 — Additional file 1. Figure S1-S6. [file 13059_2020_1934_MOESM1_ESM.pdf]

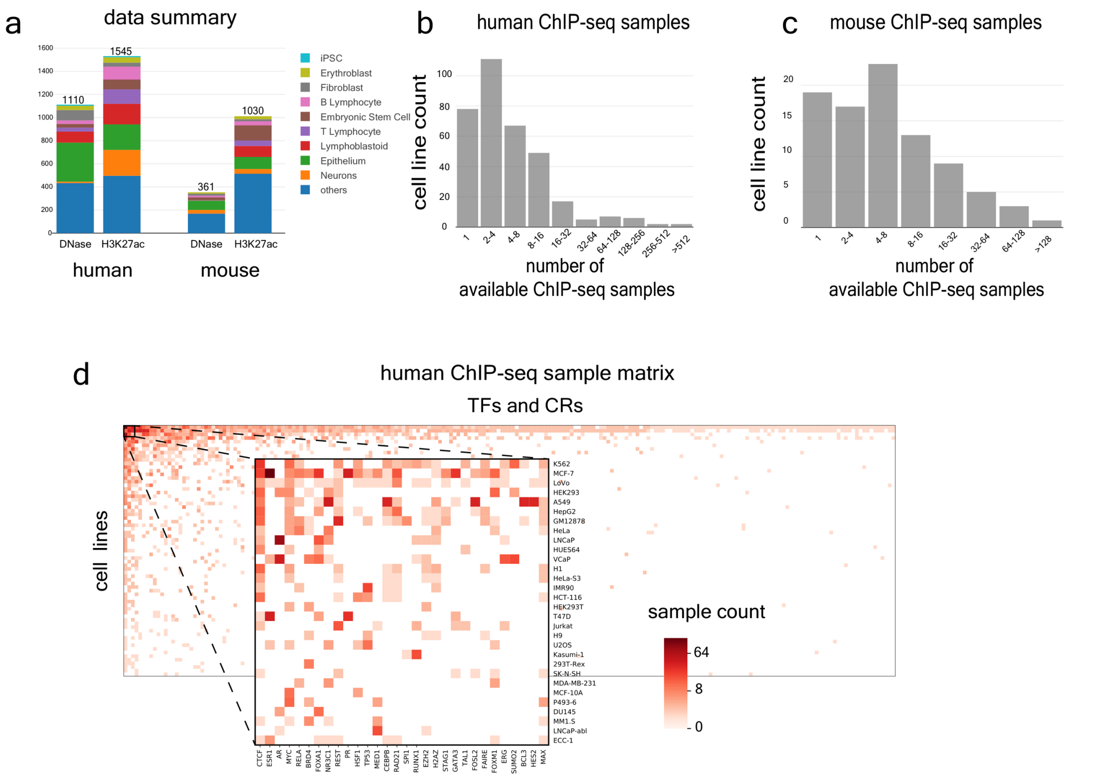

Fig. S1. Catalog of chromatin and cistrome profiles integrated in the Lisa framework. (**a**) Stacked bar plot showing the Cistrome DB (version 1) chromatin profile sample numbers in major cell types for human and mouse. Histogram showing the numbers of cell lines in which TFs in the Cistrome DB are represented by ChIP-seq samples for (**b**) human, and (**c**) mouse. (**d**) Heatmap displaying the ChIP-seq sample number for each of the TR - cell line combinations. The color represents the sample number.

a

DNase

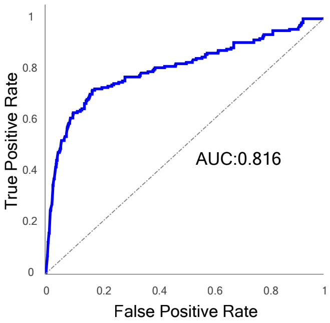

b

DNase

Sample	Model coefficient	Tissue
1	2.11	Stomach
2	0.68	Fetal Large Intestine
3	0.18	Blood
4	0.07	Retina
5	-0.01	Blood
6	-0.19	Bone Marrow
7	-0.20	Blood
8	-0.32	Mammary Gland
9	-0.65	Cord Blood
10	-0.65	Fetal Heart

c

H3K27ac

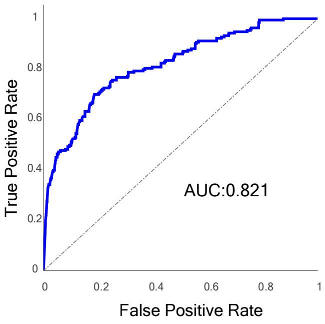

d

H3K27ac

Sample	Model coefficient	Tissue
1	0.65	Fetal Large Intestine
2	0.07	Embryonic Stem Cell
3	0.05	Cerebellum
4	0.02	Blood
5	-0.00	Blood
6	-0.07	Pancreas
7	-0.12	Blood
8	-0.12	Bone Marrow
9	-0.18	Bone Marrow
10	-0.40	Colon

Fig. S2. Lisa chromatin landscape model evaluation for the down-regulated gene set from a GATA6 knock-down experiment in gastric cancer. (**a**) ROC curve for the performance of the classifier in discriminating the target gene set from a background gene set based on DNase-seq. (**b**) DNase-seq samples selected from the Cistrome DB and the associated logistic regression coefficients. (**c**) ROC curve for the performance of the classifier in discriminating the target gene set from a background gene set based on H3K27ac ChIP-seq. (**d**) H3K27ac ChIP-seq samples selected from the Cistrome DB and the associated logistic regression coefficients.

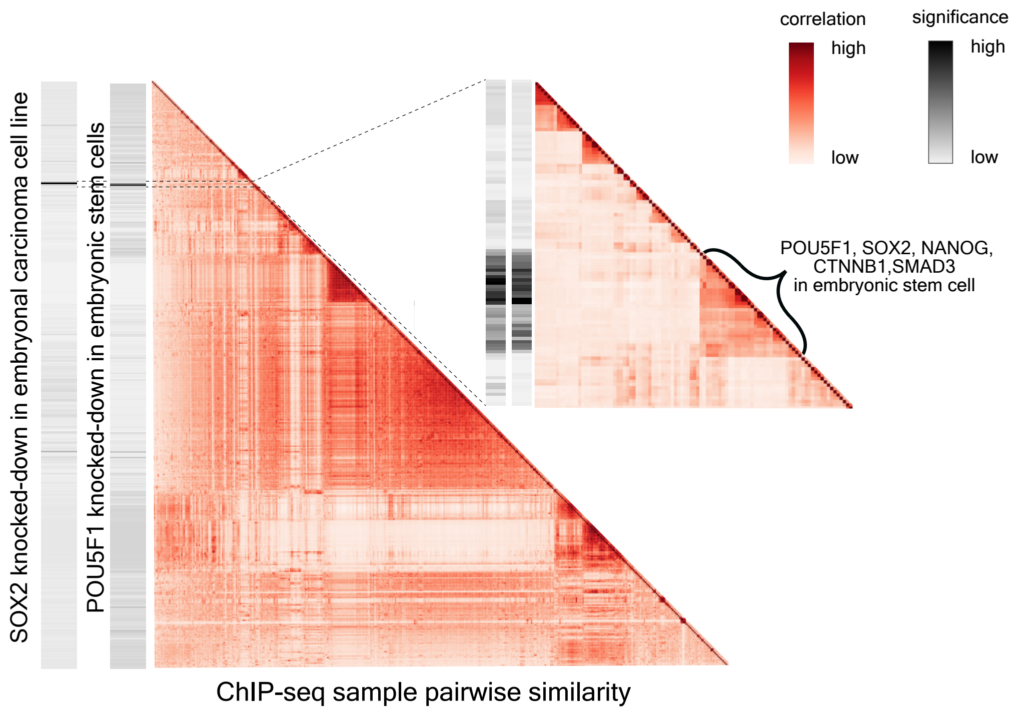

Fig. S3. Lisa predicts key transcriptional regulators and assigns significance to Cistrome DB cistromes. The large heatmap shows the hierarchical clustering of 8,471 transcriptional regulators based on peak-RP derived from Cistrome DB ChIP-seq cistromes, with color representing Pearson correlation coefficients between peak-RPs. The two bars on the left represent Lisa significance scores from SOX2 and POU5F1 perturbation experiments. A detail heatmap showing the clusters of samples enriched around the significant TFs include SOX2, POU5F1, NANOG, CTNNB1 and SMAD3.

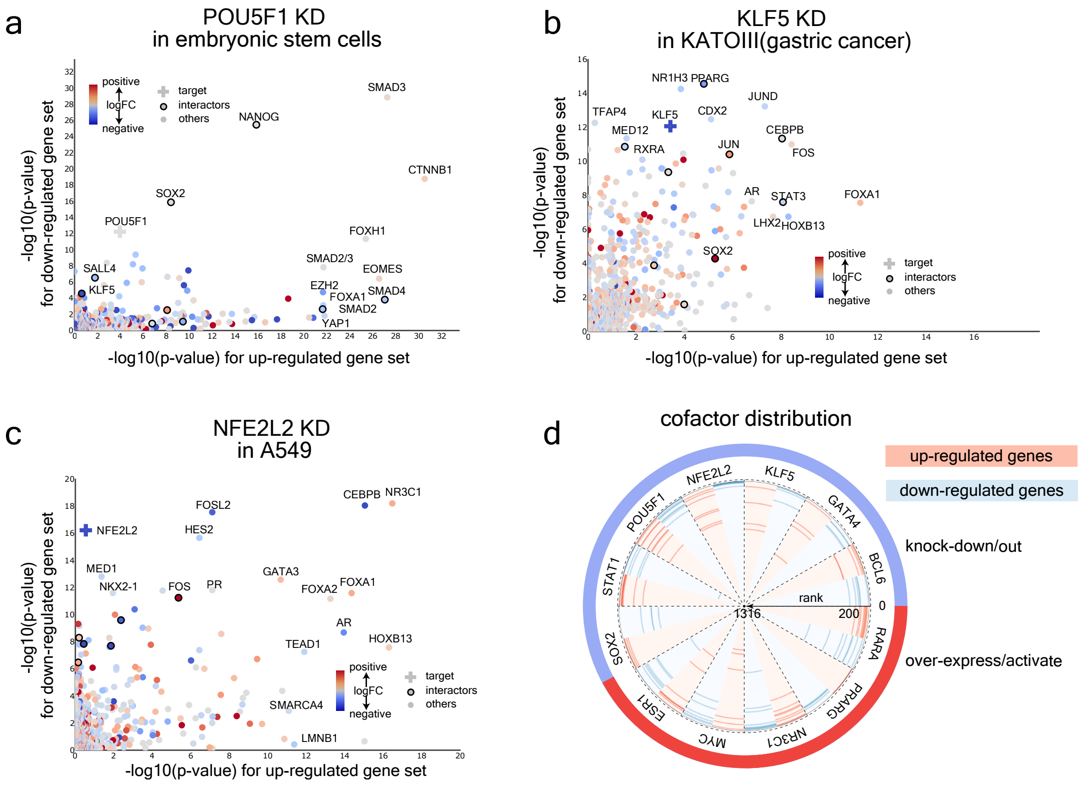

Fig. S4. Additional cases showing that Lisa can accurately infer TRs and coregulators using Cistrome DB cistromes. Lisa analysis for up- and down-regulated gene sets from (**a**) POU5F1 knock-down, (**b**) KLF5 knock-down, (**c**) NFE2L2 knock-down. The scatter plots show negative $\log_{10}$ Lisa p-values of 1316 unique transcriptional regulators for up- and down-regulated gene sets. Colors indicate $\log_2$ fold change of the TF gene expression between treatment and control conditions in the gene expression experiments. Dots outlined with a circle denote transcriptional regulators that physically interact with the target TF, which is marked with a cross. (**d**) Radar plot of cofactors discovered along with the target TFs grouped by TF perturbation. The top ranked TFs are at the perimeter and the lowest ranked are at the center. The blue and red curves represent the ranks of interactors in the Lisa analysis for down- and up-regulated gene sets, respectively.

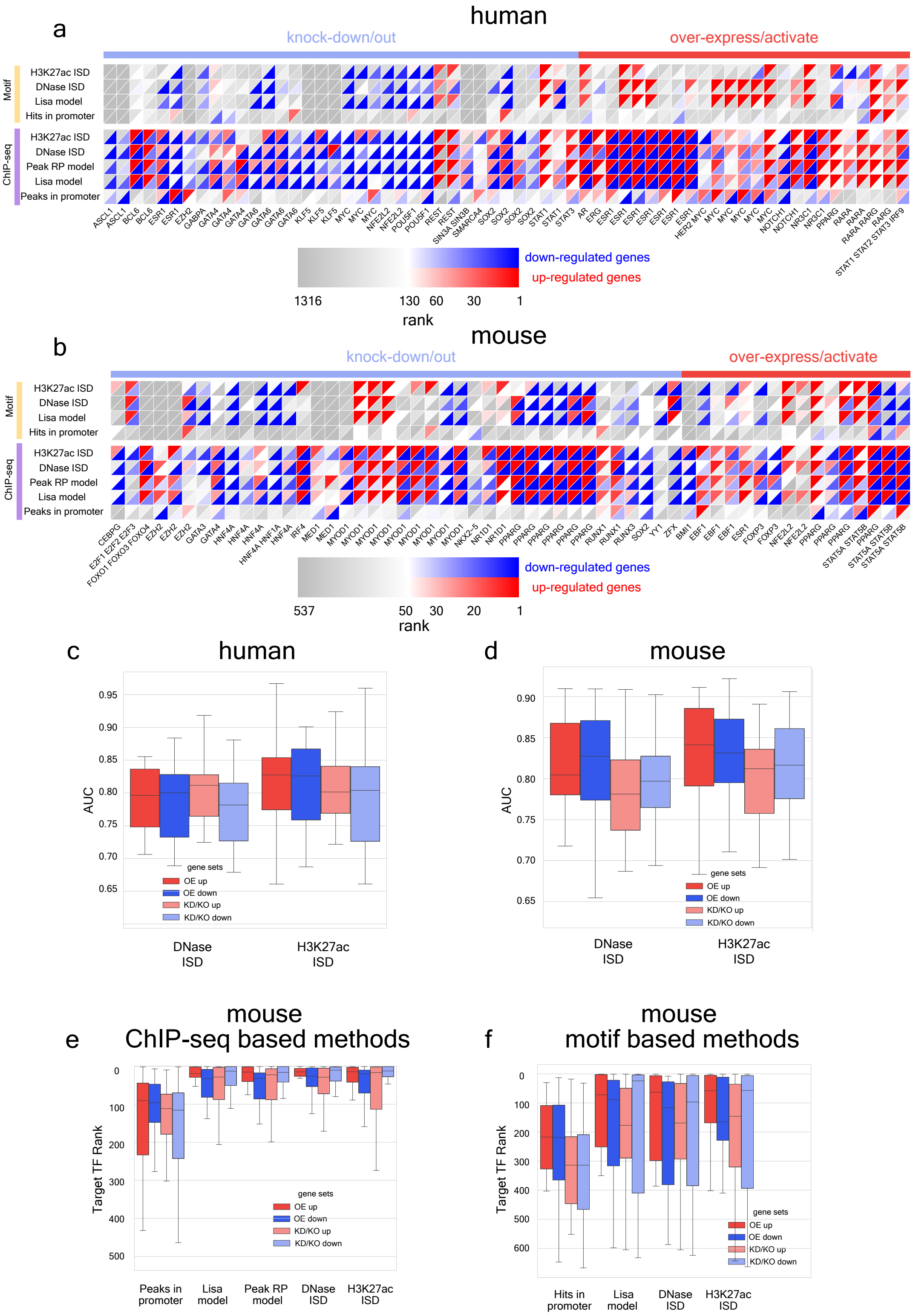

a
human
knock-down/out
over-express/activate
Motif
H3K27ac ISD
DNase ISD
Lisa model
Hits in promoter
ChIP-seq
H3K27ac ISD
DNase ISD
Peak RP model
Lisa model
Peaks in promoter
down-regulated genes
up-regulated genes
1316
130
60
30
1
rank
b
mouse
knock-down/out
over-express/activate
Motif
H3K27ac ISD
DNase ISD
Lisa model
Hits in promoter
ChIP-seq
H3K27ac ISD
DNase ISD
Peak RP model
Lisa model
Peaks in promoter
down-regulated genes
up-regulated genes
537
50
30
20
1
rank
c
human
AUC
gene sets
OE up
OE down
KD/KO up
KD/KO down
DNase ISD
H3K27ac ISD
d
mouse
AUC
gene sets
OE up
OE down
KD/KO up
KD/KO down
DNase ISD
H3K27ac ISD
e
mouse
ChIP-seq based methods
Target TF Rank
gene sets
OE up
OE down
KD/KO up
KD/KO down
Peaks in promoter
Lisa model
Peak RP model
DNase ISD
H3K27ac ISD
f
mouse
motif based methods
Target TF Rank
gene sets
OE up
OE down
KD/KO up
KD/KO down
Hits in promoter
Lisa model
DNase ISD
H3K27ac ISD

Fig. S5. Systematic application of Lisa to large-scale transcriptional regulator perturbation study reveals novel recurring regulatory patterns for both human and mouse. (**a-b**) Heatmap showing that Lisa is capable of predicting most of the TF perturbation benchmark gene sets based on cistrome profiles for (**a**) human and (**b**) mouse. Each column represents a TF activation/over-expression or knock-down/out experiment with similar experiment types grouped together. Rows represent Lisa methods based on cistromes from TR ChIP-seq data or imputed from motifs. The upper left red triangles represent the rank of the target TFs based on the analysis of the up-regulated gene sets; the lower right blue triangles represent analysis of down-regulated gene sets. The heatmap contains all of the gene sets used in the evaluation. (**c-d**) the ROC AUC metrics for the Lisa chromatin model for predicting the target gene set from TF perturbation benchmark datasets in (**c**) human and (**d**) mouse. (**e**) Boxplots showing mouse benchmark dataset performance of Lisa ChIP-seq based models and the baseline model based on TF peak counts in gene promoter regions. (**f**) Boxplots showing mouse benchmark datasets performance of Lisa motif-based methods and the baseline model based on motif hits in promoter regions.

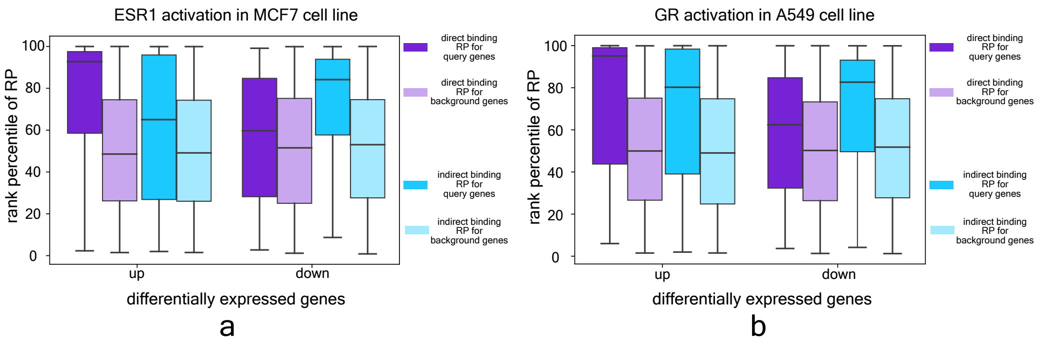

Fig. S6. Comparison of direct and indirect binding sites in ER and GR activation experiments. Direct binding sites are defined as ChIP-seq peaks with the cognate TR motif and the indirect binding sites are defined as the peaks without the motif. Peak-RPs calculated based on direct and indirect peaks are compared between query and background gene sets.
